# The Kidney Donor Profile Index (KDPI) Correlates With Histopathologic Findings in Post-reperfusion Baseline Biopsies and Predicts Kidney Transplant Outcome

**DOI:** 10.3389/fmed.2022.875206

**Published:** 2022-04-29

**Authors:** Quirin Bachmann, Flora Haberfellner, Maike Büttner-Herold, Carlos Torrez, Bernhard Haller, Volker Assfalg, Lutz Renders, Kerstin Amann, Uwe Heemann, Christoph Schmaderer, Stephan Kemmner

**Affiliations:** ^1^Department of Nephrology, School of Medicine, Klinikum Rechts der Isar, Technical University of Munich, Munich, Germany; ^2^Department of Nephropathology, Institute of Pathology, Friedrich-Alexander University (FAU) Erlangen-Nürnberg, Erlangen, Germany; ^3^School of Medicine, Institute of AI and Informatics in Medicine, Klinikum Rechts der Isar, Technical University of Munich, Munich, Germany; ^4^Department of Surgery, School of Medicine, Klinikum Rechts der Isar, Technical University of Munich, Munich, Germany

**Keywords:** kidney biopsies, living kidney donor profile index (LKDPI), ischemia/reperfusion injury, kidney transplant outcomes, expanded criteria donor (ECD), standard criteria donor (SCD), kidney donor profile index (KDPI), kidney transplantation

## Abstract

**Background:**

The increasing organ shortage in kidney transplantation leads to the necessity to use kidneys previously considered unsuitable for transplantation. Numerous studies illustrate the need for a better decision guidance rather than only the classification into kidneys from standard or expanded criteria donors referred to as SCD/ECD-classification. The kidney donor profile index (KDPI) exhibits a score utilizing a much higher number of donor characteristics. Moreover, graft biopsies provide an opportunity to assess organ quality.

**Methods:**

In a single center analysis 383 kidney transplantations (277 after deceased and 106 after living donation) performed between January 1st, 2006, and December 31st, 2016, retrospectively underwent SCD/ECD and KDPI scoring. Thereby, the quality of deceased donor kidneys was assessed by using the KDPI and the living donor kidneys by using the living KDPI, in the further analysis merged as (L)KDPI. Baseline biopsies taken 10 min after the onset of reperfusion were reviewed for chronic and acute lesions. Survival analyses were performed using Kaplan-Meier analysis and Cox proportional hazards analysis within a 5-year follow-up.

**Results:**

The (L)KDPI correlated with glomerulosclerosis (*r* = 0.30, *p* < 0.001), arteriosclerosis (*r* = 0.33, *p* < 0.001), interstitial fibrosis, and tubular atrophy (*r* = 0.28, *p* < 0.001) as well as the extent of acute tubular injury (*r* = 0.20, *p* < 0.001). The C-statistic of the (L)KDPI concerning 5-year death censored graft survival was 0.692. Around 48% of ECD-kidneys were classified as (L)KDPI<85%. In a multivariate Cox proportional hazard analysis including (preformed) panel reactive antibodies, cold ischemia time, (L)KDPI, and SCD/ECD-classification, the (L)KDPI was significantly associated with risk of graft loss (hazard ratio per 10% increase in (L)KDPI: 1.185, 95% confidence interval: 1.033–1.360, *p* = 0.025). Survival analysis revealed decreased death censored (*p* < 0.001) and non-death censored (*p* < 0.001) graft survival in kidneys with an increasing (L)KDPI divided into groups of <35, 35–85, and >85%, respectively.

**Conclusion:**

With a higher granularity compared to the SCD/ECD-classification the (L)KDPI is a promising tool to judge graft quality. The correlation with chronic and acute histological lesions in post-reperfusion kidney biopsies underlines the descriptive value of the (L)KDPI. However, its prognostic value is limited and underlines the urgent need for a more precise prognostic tool adopted to European kidney transplant conditions.

## Introduction

There is a worldwide shortage of organs suitable for kidney transplantation and especially in Germany the demand clearly exceeds the allocable organ numbers (1, 2). Therefore, rising donor age and an increased use of organs from expanded criteria donors (ECD) is recorded (3, 4).

The distinction between standard criteria donors (SCD) and ECD was introduced to grade graft quality, identifying 4 simple characteristics (age, kidney function, hypertension, and cerebrovascular death) (5). ECDs are donors who are either older than 60 years, or 50 to 59 years old and meet at least two of the following criteria: cerebrovascular death, history of hypertension, or last serum creatinine >1.5 mg/dl (Table 1). Although ECD-kidneys perform worse survival than SCD-kidneys, it could also be shown that transplantation of ECD-kidneys can be live saving compared to maintenance of hemodialysis (6–8). However, recipient's age and the increasing number of ECD-kidneys due to older donor age affects the prognostic value of the standard and expanded criteria donor classification (9).

**Table 1:** Donor and recipient characteristics used to calculate the SCD/ECD-classification, the KDPI and the LKDPI.

	**ECD**	**KDPI**	**LKDPI**
Donor associated	Age > 60 y	Age	Age
	Or	Height	BMI
	Age 50–59 y and 2 of the following:	Weight	
	Death from CVA	Arterial hypertension	Systolic blood pressure
	Arterial hypertension	Diabetes	Cigarette use
	SCr > 1.5 mg/dl	Hepatitis C	
		Cause of death	
		DBD/DCD	
		Last SCr	eGFR
		Ethnicity	Ethnicity
Transplant			AB0 incompatibility
associated			HLA-mismatches
			Weight ratio
			Biological relationship
			Sex

*BMI, Body Mass Index; CVA, cerebro-vascular accident; DBD, donation after brainstem death; DCD, donation after cardiac death; eGFR, estimated glomerular filtration rate; HLA, Human leukocyte antigen; (L)KDPI, (Living) Kidney Donor Profile Index; SCr, Serum creatinine*.

The KDPI (kidney donor profile index) is an index displayed as a cumulative percentage scale representing the risk for kidney transplant failure. For example, the graft of a donor with a KDPI of 70% has a higher predictive risk of graft failure than 70% of the grafts transplanted in the precedent year (10, 11). The KDPI is calculated from the KDRI (kidney donor risk index) which considers 10 donor-related factors including age, height, weight, history of diabetes and hypertension, serum creatinine, hepatitis C status, ethnicity, cause of death, and donation after cardiac death (Table 1). Its predictive power for transplant outcome and patient survival as well as eGFR in long term follow up after kidney transplantation has been demonstrated in several studies (12–14).

Increasing donor age is not limited to cadaveric kidneys but also affects kidneys from living donors. Furthermore, donor age is a predictor of graft function in kidney transplantation after living donation (15). The living KDPI (LKDPI) is based on the same scale as the KDPI and thus allows for graft comparison from living and deceased donors (16). Compared to the KDPI not only donor-specific parameters but also recipient- and transplant-specific variables are used in the calculation of the LKDPI such as gender, AB0 incompatibility, relationship ratio, HLA mismatches, and weight ratio (Table 1). For example, a LKDPI of 20% corresponds to the same expected graft survival as a KDPI of 20%. At the same time, the LKDPI may yield negative values, indicating a lower risk as compared to all deceased donor kidneys (16).

Here we investigated on the additional prognostic value of the (L)KDPI in SCD and ECD kidneys from a single center cohort by use of routinely taken baseline-biopsies 10 min after the onset of reperfusion. This allows for the consideration of histological graft quality including tubular injury following transplantation in the evaluation of the (L)KDPI as prognosis score in renal allografts from SCD and ECD.

## Methods

### Inclusion and Exclusion Criteria

All kidney transplantations with baseline biopsy during transplant surgery after deceased or living donation at Klinikum rechts der Isar, Munich, Germany between January 1st, 2006, and December 31st, 2016, were included in this retrospective analysis. These baseline biopsies were taken routinely 10 min after the onset of graft reperfusion by core needle (18G) biopsy as part of the clinic's internal standard of care protocol to allow for initial assessment of graft quality by baseline histology.

All patients included into this study were at least 18 years old at time of transplantation. Informed consent was obtained for using the kidney specimens retrospectively for further investigation. The local ethics committee of the Technical University of Munich, Germany had approved this retrospective analysis of the cohort (No. 178/21s). For data collection the hospital's information system, patient records, routine clinical follow-up from external nephrologists, and the Eurotransplant Network Information System (ENIS) for donor and recipient data were used. Patients were followed up until June 30th, 2017 (data lock).

Recipients with early graft failure due to perioperative (surgical and obviously non-immunological) complications were excluded from further statistical analyses.

Recipients were subclassified whether they received an organ from SCDs or ECDs according to the definition by Port et al. as written above (5).

### Classification According to (L)KDPI

For the calculation of the KDRI, ten donor characteristics (age, height, weight, ethnicity, history of hypertension and diabetes, last serum creatinine, cause of death, hepatitis C status, and donation after cardiac death) were used as guided by the Organ Procurement and Transplantation Network (OPTN) (17). In case of missing information about hypertension or diabetes, the average prevalence reported by the OPTN was used (18). Since there is no information about donor's ethnicity in the Eurotransplant system, all donors were classified as “Caucasian” according to the current German epidemiology. Using the OPTN mapping table with the scaling factor of 2017, the KDRI was translated into the KDPI score (%) (18).

The LKDPI was calculated by using donor and recipient factors such as age, eGFR, BMI, ethnicity, history of cigarette use, systolic blood pressure, sex, AB0 incompatible transplantation, relation, HLA status, and donor/recipient weight ratio (16).

Both KDPI and LKDPI were divided in groups (<35%, low risk; 35–85%, medium risk; > 85%, high risk), inspired by the OPTN. Delayed graft function (DGF) was defined as proposed by the OPTN: need for dialysis during the first week after transplantation (19).

### Primary and Secondary Endpoints

The primary endpoint was death censored transplant failure, comprising permanent need for dialysis after transplantation, including both primary non-function (apart from surgical complications) and follow-up end-stage transplant failure requiring reinstitution of dialysis. In the event of death with a functioning graft, the follow-up period was censored at date of death (20). Graft failure was assessed within 5 years after transplantation. Transplantations were censored at 5 years or at the last day of detected kidney function in follow-up examination within 5 years.

Primary non-function was defined as an initially non-working allograft with need for intermittent dialysis after transplantation, without accountable perioperative complications, and with proven organ perfusion confirmed by ultrasound examination.

The secondary endpoint was non-death censored transplant failure, which is a composite of primary non-function, follow-up end-stage transplant failure requiring the reinstitution of dialysis, and recipient death with a functioning allograft. Furthermore, we hypothesized that the (L)KDPI is associated with factors representing limited organ quality and prolonged transport, ECD, increased cold ischemia time, and histological findings in the baseline biopsy such as the histological extent of (acute) tubular injury (ATI), interstitial fibrosis and tubular atrophy (IF/TA), arteriosclerosis and glomerulosclerosis.

### Assessment of Allograft Biopsies

All biopsy specimens included in this study were retrospectively reviewed by the same experienced renal pathologist (M.B.-H.), who was blinded for clinical data. The biopsy specimens were core-needle biopsies prepared on slides containing paraffin sections (2–4 μm) that were stained with hematoxylin and eosin (HE) and periodic acid–Schiff (PAS).

Chronic lesions in the biopsies were assessed. The severity of arteriosclerosis was scored semi-quantitatively according to revised Banff Classification. The severity of IF/TA was reported as a percentage concerning the proportion of the affected cortical area in the biopsy sample. Glomerulosclerosis was expressed as a percentage of the total number of glomeruli in the biopsy (21).

ATI was scored as previously described (22) and the assessment involved apical blebbing, epithelial hydropic swelling with lucency of the cytoplasm, loss of brush border, luminal dilatation with flattening of the epithelium, cytoplasmatic vacuolization, and sloughing of tubular cells and was diagnosed whenever one or more of these histologic features were present. Thereby, the extent of ATI was categorized as “none” (0%), “mild” (<25%), “moderate” (25–50%), or “severe” (>50%) tubular injury (23).

### Statistical Analysis

Normally distributed data was summarized by mean ± standard deviation, for skewed data median and interquartile range (IQR), represented as first quartile to third quartile, are shown. Categorical data is displayed as absolute number (n) and percentage of the total number (%). Comparisons between groups of the baseline characteristics was performed by using Kruskal-Wallis and Mann-Whitney U test for non-normally distributed data, univariate ANOVA and *t*-test for normally distributed data and chi-square (χ^2^) tests for categorical data. Pearson's correlation was used to assess associations between metric, normally distributed data, Spearman rank correlation between metric and ordinal, Eta coefficient (η) between metric and nominal data, and the χ^2^ -test (ϕ) between ordinal and nominal scaled variables.

Kaplan-Meier analysis, univariate and multivariate Cox proportional-hazards analysis, and log-rank tests were used to examine the association between the SCD/ECD-classification as well as the (L)KDPI and the primary and secondary endpoint. Univariate and multivariate Cox proportional-hazards analyses were calculated with the 5-year follow-up values.

For estimation of hazard ratios, Cox proportional-hazards models were fitted to the data. Those multivariate models included recipient and donor associated risk factors from univariate analysis for the primary endpoint (death censored transplant failure). The (L)KDPI score was included in a Cox proportional-hazards analysis as a continuous variable. All tests were performed two-sided using a significance level of α = 0.05. C-statistics (24) were estimated using the concordance() function provided in the survival package of R (25, 26).

Statistical elaboration was performed using the software programs “IBM SPSS Statistics” version 25 (IBM Corp., NY, USA) and “R” version 3.4.4 (R development team, Vienna, Austria). In addition, GraphPad Prism, version 7.0 (Graph-Pad Software) was used for data presentation.

## Results

### Patients

**Figure 1:**
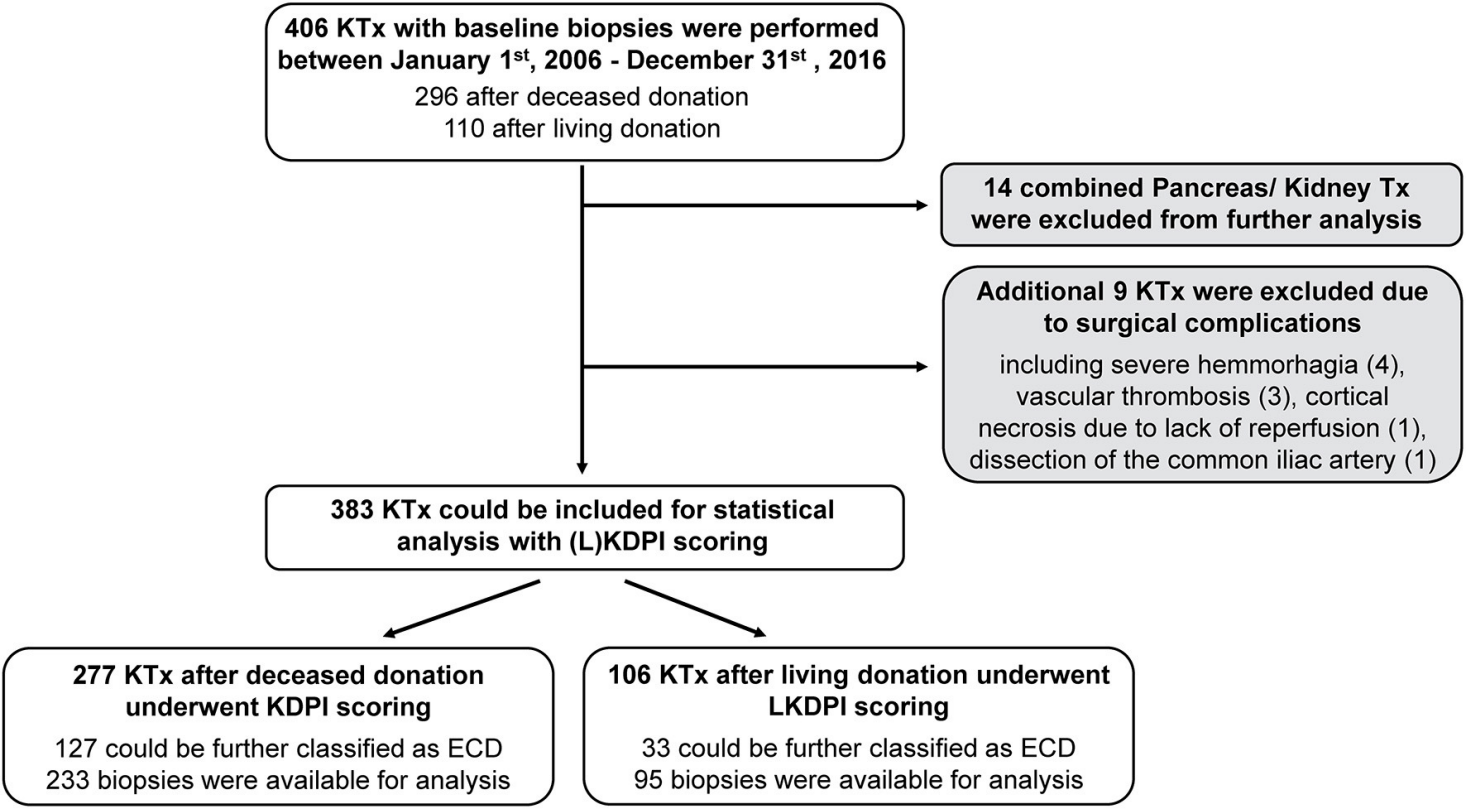
Study population. Flowchart representing the evaluation process of kidney transplantations for statistical analysis and histological judgement of baseline biopsies. Expanded Criteria Donor (ECD); (Living) Kidney Donor Profile Index (L)KDPI; kidney transplantation KTx.

In total, 406 potential kidney transplantations (Figure 1) which underwent baseline biopsy were performed between January 1st, 2006, and December 31st, 2016, at Klinikum rechts der Isar, Munich. Of these, 14 underwent combined kidney-pancreas transplantation and were therefore excluded from statistical analysis, as well as nine transplantations with early graft loss due to perioperative (surgical) complications.

Of these 383 remaining transplantations, 277 underwent KDPI scoring according to deceased donation and 106 transplantations underwent LKDPI scoring after living donation. During the observation period five patients were transplanted twice due to early failure of first kidney transplant. Further, of the 277 deceased donations 150 (54%) were classified as SCD and 127 (46%) as ECD. In living donations 73 (69%) were classified as SCD and 33 (31%) as ECD. Detailed baseline demographics are presented in Table 2.

**Table 2:** Demographic and clinical characteristics of donors and recipients in the total cohort and in kidney transplantations after living or deceased donation.

**Characteristics**	**Total**	**Living**	**Deceased**	***p*-value**
Number, *n* (%)	383 (100)	106 (28)	277 (72)	
Living donors, *n* (%)	106 (28)	106 (100)	0 (0)	<0.001
**Donor associated**				
(L)KDPI	54 (27; 83)	28 (8; 49)	67 (38; 89)	**<0.001**
Female, *n* (%)	172 (45)	62 (59)	110 (40)	**0.001**
Age (years)	53 ± 15	54 ± 11	53 ± 16	0.313
BMI (kg/m^2^)	27 ± 5	27 ± 4	27 ± 5	0.451
Cause of death (*n*)	277	0	277	
Trauma	63 (23)		63 (23)	
CVA	160 (58)		160 (58)	
Other	54 (20)		54 (20)	
**History of**				
hypertension	154 (41)	38 (36)	116 (42)	0.217
diabetes	38 (10)	0 (0)	38 (14)	**<0.001**
last SCr (mg/dl)	0.9 (0.7; 1.1)	0.8 (0.7; 0.9)	0.9 (0.7; 1.3)	**0.004**
**Transplant associated**				
HLA-mismatch	4 (3; 5)	4 (3; 5)	4 (3; 5)	0.154
CIT (h)	8 (2; 13)	2 (2; 2)	11 (8; 15)	**<0.001**
WIT (min)	20 (20; 22)	20 (20; 20)	20 (18; 30)	0.726
**Recipient associated**				
Female, *n* (%)	134 (35)	37 (35)	97 (35)	0.984
Age (years)	52 ± 13	47 ± 13	55 ± 12	**<0.001**
BMI (kg/m^2^)	25 ± 5	25 ± 5	25 ± 5	0.952
Caucasian	377(98)	105 (99)	272 (98)	0.362
First transplantation	318 (83)	97 (92)	221 (80)	**0.006**
Induction therapy	89 (23)	25 (24)	64 (23)	0.171
**Reason for ESKD**				
Glomerulonephritis	117 (31)	34 (32)	83 (30)	0.688
Diabetes	37 (10)	9 (9)	28 (10)	0.632
Hypertension	57 (15)	15 (14)	42 (15)	0,803
Other	172 (45)	48 (45)	124 (45)	0.734
Duration of dialysis (months)	51 (19; 86)	5 (0; 17)	70 (43; 93)	**<0.001**
**Immunosuppression**				
Glucocorticoids	382 (100)	106 (100)	277 (100)	
CNI	382 (100)	106 (100)	277 (100)	
Tacrolimus	296 (77)	99 (93)	197 (71)	**<0.001**
CCI Score	2 (2,4)	2 (2,3)	3 (2,4)	**0.012**
**Results**				
**Transplant failure**				
After 1 year	25 (7)	1 (1)	24 (9)	**0.006**
After 3 years	38 (10)	5 (5)	33 (12)	**0.035**
After 5 years	47 (12)	7 (7)	40 (14)	**0.037**
**Death with functioning transplant**				
After 1 year	16 (4)	1 (1)	15 (5)	0.050
After 3 years	30 (8)	2 (2)	28 (10)	**0.007**
After 5 years	34 (9)	2 (2)	32 (12)	**0.003**
Delayed graft function	124 (32)	16 (15)	108 (41)	**<0.001**
Primary non function	14 (4)	1 (1)	13 (5)	0.080
Patients with rejections within 1 year	102 (27)	34 (32)	68 (25)	0.136
**eGFR (ml/min/1,73 m^2^)**				
After 3 years	48 (36; 64)	58 (42; 71)	44 (35; 61)	**0.002**

*n (%) for categorical data, mean ± standard deviation for normally distributed data, median [interquartile range] for non-parametric data. Comparison between living and deceased groups by χ2 for categorical data, independent t-test for normally distributed and Mann-Whitney U test for non-parametric data. BMI, Body Mass Index; CCI Score, Charlson Comorbidity Score; CIT, cold ischemia time; eGFR, estimated glomerular filtration rate; ESKD, end stage kidney disease; HLA, Human leukocyte antigen; (L)KDPI, (Living) Kidney Donor Profile Index; SCr, Serum creatinine; WIT, warm ischemia time; CVA, cerebro-vascular accident*.

*Statistically significant p-values are printed in bold*.

Since in Germany non-heart-beating kidney donation is not possible all deceased donations in this cohort were donations after brainstem death (DBD) and will be referred to as such.

Of 383 initially taken biopsy samples, 54 specimens were not available for further analysis due to poor specimen quality, e.g., insufficient cortical tissue or autolysis. The median follow-up time for recipients at the time of data extraction from the clinical follow-up database (data lock: June 30th, 2017) was 4.8 (0.1–11.4) years. During observation, three patients were lost to follow-up and censored: one patient after deceased donation after 54 days and two patients after living donation (after 342 and 428 days).

### Renal Graft Outcomes

Within 5 years after transplantation 47 patients suffered from transplant failure and 34 patients died with a functioning allograft. Of these, 8 patients with transplant failure had a transplant with a KDPI or LKDPI [(L)KDPI] of <35%, 17 patients of 35–85%, and 22 patients of more than 85%. Primary non-function occurred in 1 kidney transplant with a (L)KDPI of <35%, in 6 transplants of 35–85% and in 7 transplants of more than 85% (*p* = 0.018). Of these, only one patient received a living donation (LKDPI 35–85%). There also were significant differences in the eGFR of renal grafts 3 years after transplantation in the three categories (L)KDPI <35, 35–85, and >85% with eGFRs of 61 ml/min/1.73 m^2^, 45 ml/min/1.73 m^2^, and 39 ml/min/1.73 m^2^ (*p* < 0.001), respectively. Focusing on transplantation after deceased donation only, the eGFR after 3 years showed comparable values in the three (L)KDPI categories: 65 ml/min/1.73 m^2^, 45 ml/min/1.73 m^2^, and 38 ml/min/1.73 m^2^ (*p* < 0.001). No significant differences were present in the number of rejections 1 year after transplantation between the (L)KDPI groups, neither for all transplantations nor within the recipients after deceased donation. Average dialysis vintage was not different between the 3 KDPI-groups of kidney transplantations after DBD. Significant differences were also present in 3 year-eGFR and PNF for transplants divided by the SCD/ECD-criteria, whereas there was no significant difference for biopsy proven rejections (BPRs) as shown in Table 3.

**Table 3:** Demographic and clinical characteristics of donors and recipients, divided in SCD/ECD and (L)KDPI groups.

**Characteristics**	**SCD**	**ECD**	***p*-value**	**(L)KDPI-score**			***p*-value**
				** <35**	**35–85**	**>85**	
Number, *n* (%)	223 (58)	160 (42)		127 (33)	171 (45)	85 (22)	
Living donors, *n* (%)	73 (33)	33 (21)		61 (48)	44 (26)	1 (1)	
**Donor associated**							
(L)KDPI (%)	31 (14; 53)	87 (70; 95)	**<0.001**	16 (3; 27)	58 (50; 72)	95 (90; 98)	**<0.001**
ECD				7 (6)	70 (41)	83 (98)	**<0.001**
Female, *n* (%)	99 (44)	73 (46)	0.811	46 (36)	94 (55)	32 (38)	**0.002**
Age (years)	44 ± 12	66 ± 7	**<0.001**	41 ± 13	55 ± 9	69 ± 10	**<0.001**
BMI (kg/m^2^)	27 ± 5	28 ± 4	**0.014**	26 ± 5	27 ± 5	28 ± 4	0.095
Cause of death (*n*)	150	127		66	127	84	
Trauma	52 (35)	11 (9)	**<0.001**	39 (31)	17 (10)	7 (8)	**<0.001**
CVA	63 (42)	97 (76)	**<0.001**	6 (5)	87 (51)	67 (80)	**<0.001**
Other	35 (23)	19 (15)	0.289	21 (17)	23 (13)	10 (12)	0.587
**History of**							
Hypertension	55 (25)	99 (62)	**<0.001**	18 (14)	80 (47)	56 (66)	**<0.001**
Diabetes	14 (6)	24 (15)	**0.005**	0 (0)	18 (11)	20 (24)	**<0.001**
Last SCr (mg/dl)	0.8 (0.7; 1.1)	0.9 (0.7; 1.2)	0.076	0.8 (0.7; 1.0)	0.8 (0.7; 1.1)	1.0 (0.8; 1.3)	**0.002**
**Transplant associated**							
HLA-mismatch	3 (3; 4)	4 (3; 5)	**<0.001**	3 (2; 4)	4 (3; 5)	5 (4; 5)	**<0.001**
CIT (h)	8 (2; 13)	8 (4; 14)	0.231	4 (2; 12)	8 (3; 14)	10 (6; 16)	**<0.001**
WIT (min)	20 (20; 20)	20 (20; 30)	0.782	20 (20; 20)	20 (20; 20)	20 (20; 30)	0.062
**Recipient associated**							
Female, *n* (%)	78 (35)	56 (35)	0.996	51 (40)	55 (32)	28 (33)	0.325
Age (years)	48 ± 12	59 ± 12	**<0.001**	46 ± 13	52 ± 11	63 ± 10	**<0.001**
BMI (kg/m^2^)	25 ± 5	26 ± 5	0.039	24 ± 5	26 ± 5	25 ± 4	**0.048**
**Reason for ESKD**							
Glomerulonephritis	68 (30)	49 (31)	0.978	45 (35)	47 (27)	25 (29)	0.327
Diabetes	19 (9)	18 (11)	0.372	8 (6)	21 (12)	8 (9)	0.224
Hypertension	35 (16)	22 (14)	0.598	17 (13)	24 (14)	16 (19)	0.506
Other	101 (45)	71 (44)	0.859	57 (45)	79 46)	49 (58)	0.222
Duration of dialysis (months)	51 (13; 87)	50 (28; 86)	0.563	25 (4; 80)	68 (26; 92)	49 (33; 69)	**<0.001**
CCI Score	2 (2; 3)	3 (2; 4)	**0.004**	2 (2; 3)	2 (2; 3)	3 (2; 4)	**0.001**
**Results**							
**Transplant failure**							
After 5 years	16 (7)	31 (19)	**<0.001**	8 (7)	17 (10)	22 (26)	**<0.001**
**Death with functioning transplant**							
After 5 years	192 (86)	110 (69)	0.081	3 (2)	15 (9)	16 (19)	**<0.001**
Delayed graft function	67 (30)	57 (36)	0.139	24 (19)	66 (39)	34 (40)	**<0.001**
Primary non function	4 (2)	10 (6)	**0.022**	1 (1)	6 (4)	7 (8)	**0.018**
Patients with rejections within 1 year	52 (23)	50 (31)	0.097	29 (23)	48 (28)	25 (29)	0.483
**eGFR (ml/min/1,73 m^2^)**							
After 3 years	54 (40; 71)	41 (31; 51)	**<0.001**	57 (45; 74)	48 (37; 61)	35 (30; 45)	**<0.001**

*n (%) for categorical data, mean ± standard deviation for normally distributed data, median [interquartile range] for non-parametric data. Comparison between SCD and ECD by χ2 for categorical data, independent t-test for normally distributed and Mann-Whitney U test for non-parametric data. Comparison of (L)KDPI groups by χ2 for categorical data, ANOVA for normally distributed or Kruskal-Wallis test for non-parametric data. BMI, Body Mass Index; CCI Score, Charlson Comorbidity Score; CIT, cold ischemia time; CVA, cerebro-vascular accident; ECD, Expanded Criteria Donor; eGFR, estimated glomerular filtration rate; ESKD, endstage kidney disease; HLA, Human leukocyte antigen; (L)KDPI, (Living) Kidney Donor Profile Index; SCD, Standard Criteria Donor; SCr, Serum creatinine; WIT, warm ischemia time*.

*Statistically significant p-values are printed in bold*.

**Figure 2:**
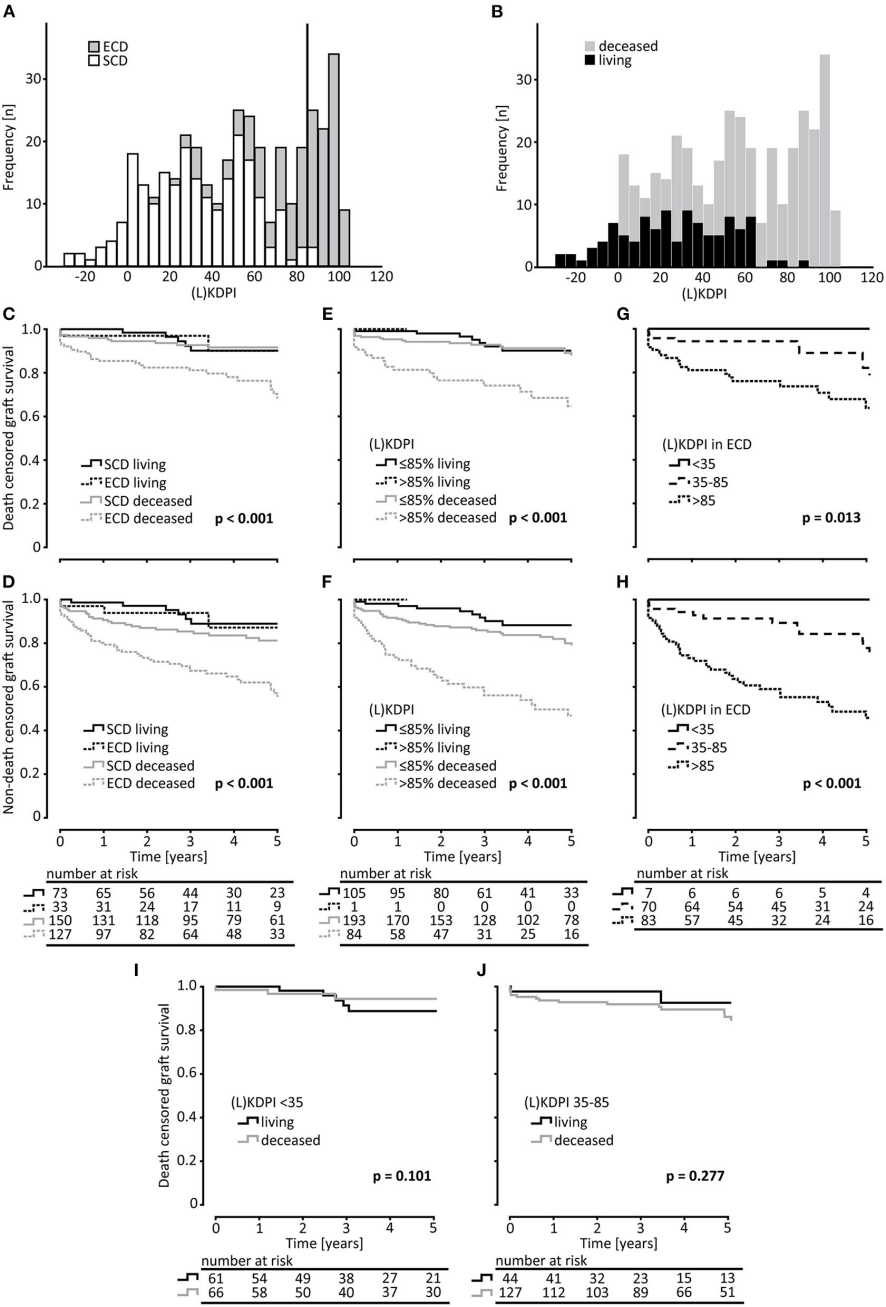
Survival analysis of kidney transplantations rated by the ECD-criteria and the (L)KDPI. **(A)** Histogram of the distribution of Standard Criteria Donor (SCD) and Expanded Criteria Donors (ECD) in (Living) Kidney Donor Profile Index [(L)KDPI] rated transplantations. **(B)** Histogram of the distribution of living and deceased transplantations in (Living) Kidney Donor Profile Index [(L)KDPI] rated transplantations. On the x-axis the transplantations are divided into groups of (L)KDPI-increase = 5. **(C–F)** Kaplan-Meier estimates for death censored graft survival and non-death censored graft survival for SCD vs. ECD and (L)KDPI ≤85% and >85% of living and deceased donation. **(G,H)** Kaplan-Meier estimates for death censored graft survival and non-death censored graft survival of ECD-kidneys for survival of (L)KDPI groups of <35, 35–85, and >85. Living and deceased donation was pooled for this analysis. **(I,J)** Kaplan-Meier estimates for death censored graft survival comparing living and deceased donation for (L)KDPI <35% and 35–85%. Log-rank testing was used for calculation of each *p*-value.

Transplant failure and death with functioning graft increased significantly with a (L)KDPI >85% compared to (L)KDPI <35% and 35–85% (Figures 2E,F). Graft loss at 5 years was 8/127 (Kaplan-Meyer estimator 0.92) for (L)KDPI <35%, 17/171 (Kaplan-Meyer estimator 0.88) for (L)KDPI 35–85%, and 22/85 (Kaplan-Meyer estimator 0.65) for (L)KDPI >85% respectively (*p* < 0.001). Nonetheless, average death censored graft survival in the high-risk group was still 7.5 years (±1.2 years). Mean death censored graft survival time was 9.3 years (±0.7 years) in the medium- and 10.3 years (±1.2 years) in the low-risk group. In transplantation from DBDs it was 9.1 (±0.8 years) and 10.6 years (±0.6 years), respectively. The median LKDPI in transplantations after living donation was 28 (IQR: 8, 59) whereas the median KDPI after deceased donation was 67 (IQR: 37, 89; *p* < 0.001, Figure 2B). The eGFR of kidneys with (L)KDPI >85% 3 years after transplantation was only 9 ml/min/1.73 m^2^ below the overall average. DGF occurred in 24/127 (19%) transplanted kidneys with (L)KDPI of <35%, in 66/171 (39%) transplanted kidneys with (L)KDPI of 35–85%, and in 34/85 (40%) transplanted kidneys with (L)KDPI of >85% (*p* < 0.001). The recipient's Charlson Comorbidity Index correlated with the (L)KDPI of all recipients irrespective of living or deceased donation (*r* = 0.18, *p* < 0.001, **Figure 4A**).

### Predictive Value of the (L)KDPI

The (L)KDPI as a continuous variable was significantly associated with death censored graft survival (HR per 10% increase in (L)KDPI: 1.197, 95% CI: 1.085–1.320, *p* < 0.001) and non-death censored graft survival (HR per 10% increase in (L)KDPI: 1.221, 95% CI: 1.129–1.231, *p* < 0.001). Likewise, this was applicable to the KDPI after deceased donation for death censored graft survival (HR per 10% increase in (L)KDPI: 1.297, 95% CI: 1.153–1.459, *p* < 0.001) and non-death censored graft survival (HR per 10% increase in (L)KDPI: 1.259, 95% CI: 1.164–1.361, *p* < 0.001) but not for the LKDPI in living donation (Table 4). As dichotomous variable the SCD/ECD-classification reaches a greater association compared to the continuous (L)KDPI for death censored graft survival (HR 2.223, 95% CI: 1.509–3.275, *p* < 0.001) and non-death censored graft survival (HR 2.602, 95% CI: 1.539–4.397, *p* < 0.001), respectively. Table 4 shows the HR for previously identified factors influencing kidney transplantation outcomes for death censored and non-death censored graft survival.

**Table 4:** Univariate Cox proportional hazards models for 5-year death censored and non-death censored graft survival with hazard ratios (HR) and 95% confidence intervals (CI) for donor, recipient and transplant associated factors.

	**Death censored graft survival HR (95% CI)**	***p*-value**	**Non-death censored graft survival HR (95% CI)**	***p*-value**
**Donor associated**				
(L)KDPI	1.197 (1.085–1.320)	**<0.001**	1.221 (1.129–1.231)	**<0.001**
KDPI	1.297 (1.153–1.459)	**<0.001**	1.259 (1.164–1.361)	**<0.001**
LKDPI	0.852 (0.660–1.099)	0.229	0.951 (0.782–1.157)	0.659
ECD	2.602 (1.539–4.397)	**<0.001**	2.223 (1.509–3.275)	**<0.001**
Age	1.038 (1.018–1.059)	**<0.001**	1.039 (1.024–1.055)	**<0.001**
Gender (f)	1.019 (0.610–1.702)	0.943	1.271 (0.866–1.864)	0.221
Height	0.995 (0.973–1.017)	0.660	0.989 (0.975–1.003)	0.115
Weight	1.008 (0.993–1.024)	0.308	1.000 (0.988–1.013)	0.949
**History of**				
Hypertension	2.347 (1.381–3.988)	**0.002**	1.656 (0.114–2.459)	**0.012**
Diabetes	4.471 (2.462–8.119)	**<0.001**	2.973 (1.818–4.863)	**<0.001**
Smoking	0.508 (0.264–0.979)	**0.043**	0.400 (0.243–0.659)	**<0.001**
Cause of death: CVA	1.888 (1.026–3.474)	**0.041**	1.950 (1.240–3.067)	**0.004**
Last SCr	0.820 (0.492–1.366)	0.445	0.818 (0.557–1.201)	0.305
**Recipient associated**				
Age	1.023 (1.001–1.046)	**0.036**	1.047 (1.028–1.066)	**<0.001**
BMI	1.050 (0.999–1.104)	0.057	1.015 (0.976–1.056)	0.458
Gender (f)	0.929 (0.545–1.585)	0.787	0.732 (0.483–1.111)	0.143
CCI	1.047 (0.832–1.318)	0.696	1.326 (1.142–1.540)	**<0.001**
**Reason for ESKD**				
Glomerulonephritis	0.717 (0.399–1.288)	0.266	0.618 (0.392–0.975)	**0.039**
Diabetes	1.639 (0.778–3.456)	0.194	2.014 (1.198–3.386)	**0.008**
Hypertension	0.511 (0.204–1.279)	0.152	1.137 (0.684–1.890)	0.621
Duration of dialysis	1.005 (0.998–1.011)	0.145	1.003 (0.998–1.008)	0.259
**Transplant associated**				
Living vs. deceased donation	1.745 (0.882–3.452)	0.109	2.150 (1.243–3.719)	**0.006**
CIT	1.022 (0.981–1.065)	0.297	1.042 (1.011–1.074)	**0.007**
WIT	1.011 (1.002–1.019)	**0.011**	1.009 (1.002–1.017)	**0.012**
Number of HLA-mismatches	1.318 (1.090–1.595)	**0.004**	1.263 (1.099–1.452)	**0.001**
PRA	1.013 (1.006–1.021)	**0.001**	1.007 (1.000–1.014)	**0.039**
DGF	2.138 (1.191–3.839)	**0.011**	1.514 (0.996–2.302)	0.052
Number of BPR in first year	2.021 (1.607–2.541)	**<0.001**	1.802 (1.483–2.190)	**<0.001**
Number of all BPR	0.613 (0.421–0.894)	**0.011**	0.670 (0.506–0.886)	**0.005**
eGFR after 3 years	0.957 (0.931–0.983)	**0.001**	0.967 (0.949–0.986)	**0.001**

*HR and CI were calculated per 10% increase of the (L)KDPI. BMI, Body Mass Index; BPR, biopsy-proven rejection; CCI, Charlson Comorbidity Index; CIT, cold ischemia time; CVA, cerebro-vascular accident; DGF, delayed graft function; eGFR, estimated glomerular filtration rate; ESKD, end stage kidney disease; HLA, Human leukocyte antigen; (L)KDPI, (Living) Kidney Donor Profile Index; PRA, panel-reactive antibody; SCr, Serum creatinine; TX, transplantation; WIT, warm ischemia time*.

*Statistically significant p-values are printed in bold*.

The estimated C-statistics of long-term death censored graft survival (5 years) was 0.692 (±0.042) for the (L)KDPI alone and 0.714 (±0.05) if IF/TA in the post reperfusion biopsy was included into the model. On the other hand, donor age alone also yielded a C-statistic of 0.662 (±0.043). The C-statistic of 1-year prediction of death censored graft survival was 0.775 (±0.046) for the (L)KDPI alone and 0.772 (±0.056) for (L)KDPI + IF/TA. In comparison, the C-statistics of donor age considering events within 1 year was 0.715 (±0.053).

Nonetheless, in a multivariate Cox-regression model the (L)KDPI was significantly associated with death censored graft survival if ECD-status, panel reactive antibodies (PRA) and cold ischemia time (CIT) were considered in the model (HR per 10% increase in (L)KDPI: 1.185, 95% CI: 1.033–1.360, *p* = 0.025, Table 5). If the same model was applied to DBD kidneys only, the significant association with the KDPI was increased (HR per 10% increase in KDPI: 1.323, 95% CI: 1.088–1.610, *p* = 0.006). To exclude the bias of assignment of better kidney grafts to younger patients, we also fitted a Cox-regression for death censored graft survival to the data with the KDPI (only DBD), ECD-status, PRA, and the recipient's age as independent variables. Here also the KDPI showed a significant association (HR per 10% increase in KDPI: 1.336, 95% CI: 1.077–1.658, *p* = 0.008). Interestingly, in all models including PRA, PRA also had a HR statistically significant from one, indicating its independent association from all other factors investigated on death censored graft survival. To investigate the known highly important association between the primary outcome and donor age, a Cox-regression was calculated for the (L)KDPI and donor age. In this model neither parameters could prove a significant association.

**Table 5:** Multivariate Cox-regression model for death censored graft survival with hazard ratios (HR) and 95% confidence intervals (CI) including prognostic factors for reduced graft survival.

**Variables**	**Model 1**		**Model 2**		**Model 3**		**Model 4**	
	**HR (95% CI)**	***p*-value**	**HR (95% CI)**	***p*-value**	**HR (95% CI)**	***p*-value**	**HR (95% CI)**	***p*-value**
(L)KDPI	1.139 (0.993; 1.306)	0.066	1.185 (1.033; 1.360)	**0.025**	1.323 (1.088; 1.610)	**0.006**	1.336 (1.077; 1.658)	**0.008**
ECD			1.564 (0.700; 3.497)	0.276	1.118 (0.436; 2.865)	0.817	1.091 (0.430; 2.764)	0.855
PRA			1.015 (1.007; 1.023)	**<0.001**	1.015 (1.007; 1.023)	<**0.001**	1.015 (1.006; 1.024)	**0.001**
Recipient age							0.998 (0.970; 1.027)	0.896
Donor age	1.016 (0.986; 1.047)	0.293						
CIT			1.007 (0.959; 1.057)	0.787	1.009 (0.955; 1.066)	0.751		

*Models 1 and 2 include all 383 kidney transplantations and models 3 and 4 only transplantations after deceased donation. HR and CI were calculated per 10% increase of the (L)KDPI. CIT, cold ischemia time; ECD, Expanded Criteria Donor; (L)KDPI, (Living) Kidney Donor Profile Index; PRA, panel reactive antibodies*.

*Statistically significant p-values are printed in bold*.

### Accuracy of (L)KDPI and the SCD/ECD-Classification

Although there was a statistically significant association between the LKDPI graft survival in living donation, it was possible to judge survival of living and DBD grafts with the KDPI (Figures 2E,F). Comparing death censored graft survival of all living and all DBD grafts in our cohort, both KDPI and LKDPI showed no significant differences in the two superior categories <35% and 35–85% (Figures 2I,J). Thus, we investigated the influence of (L)KDPI >85% on death censored graft survival compared to transplantations of (L)KDPI of ≤85% (<35% and 35–85% combined). As categorical variable (L)KDPI ≤85% and >85% the HR of death censored graft survival was 3.205 (95% CI 1.888–5.442, *p* < 0.001) for all transplantations and 2.981 (95% CI 1.682–5.283, *p* < 0.001) after DBD, respectively. Since there was only one living donation with an LKDPI >85%, we were not able to perform statistical analyses in this group. Thus, we decided to pool living and deceased donor kidneys into one analysis. Although 31% of the living donations were classified as ECD, there was no relevant difference between survival of ECD and SCD kidneys in living donations (Figures 2C,D).

In total, 77 kidney grafts with a (L)KDPI of ≤85% were classified as ECD whereas only 2 renal grafts >85% were classified as SCD (Figure 2A). Survival of ECD-kidneys divided into the 3 (L)KDPI groups showed significant differences for death censored and non-death censored graft survival (Figures 2G,H).

### Correlation of (L)KDPI With the Histology of Post-reperfusion Graft Baseline Biopsies and Ischemia-Reperfusion Injury

**Figure 3:**
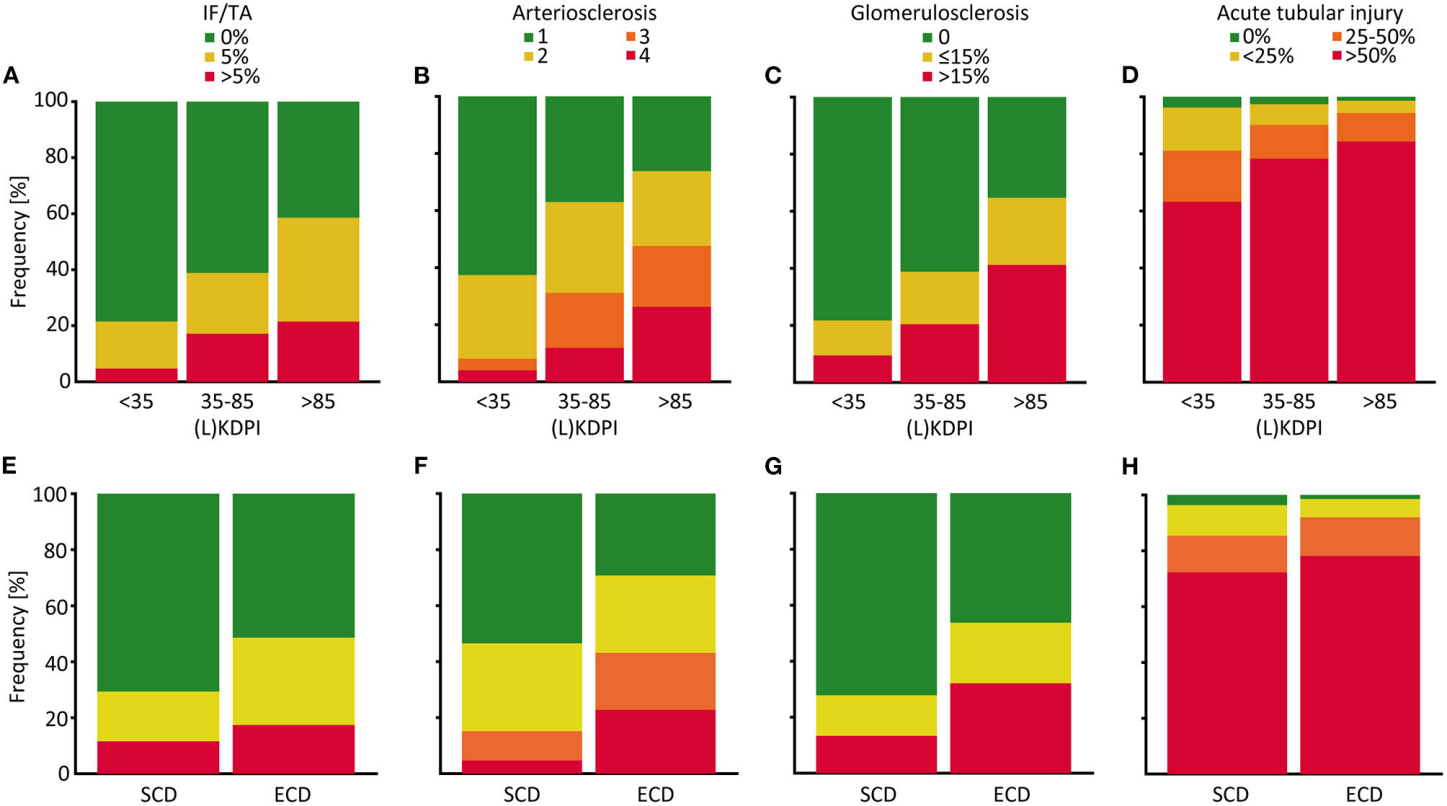
Distribution of histological properties of post-reperfusion biopsies depending on the (L)KDPI of kidney transplantations after living and deceased donation. Percent stacked column chart of the amount of interstitial fibrosis and tubular atrophy (IF/TA), arteriosclerosis, glomerulosclerosis and acute tubular injury subdivided into (Living) Kidney Donor Profile Index [(L)KDPI] <35, 35–85, and >85% **(A–D)** or Standard Criteria Donor (SCD) and Expanded Criteria Donor (ECD) **(E–H)**. Kidney graft tissue was taken 10 min after the onset of reperfusion by 18G core needle biopsy. Histological evaluation was performed by one renal pathologist blinded for clinical data. A semi-quantitative score according to the Banff Classification was used to assess arteriosclerosis. IF/TA, glomerulosclerosis, and acute tubular injury are shown as percentage of the entire area used for histological investigation.

To assess correlation of the (L)KDPI with the quality of the transplanted kidneys more accurately we included the histopathological findings in post-reperfusion biopsies into the statistical analyses as described above. Glomerulosclerosis, arteriosclerosis, and IF/TA as chronic lesions and ATI as renal hallmark of acute injury were histologically evaluated. Naturally, the extent of the chronic parameters increases with an increasing (L)KDPI (Figures 3A–C). Fittingly, we found a significant correlation between these parameters and the (L)KDPI (glomerulosclerosis *r* = 0.30, *p* < 0.001; arteriosclerosis *r* = 0.33, *p* < 0.001; IF/TA *r* = 0.28, *p* < 0,001). This was most likely due to the high number of deceased donations as only arteriosclerosis turned out to significantly correlate in living donations (glomerulosclerosis *r* = 0.03, *p* = 0.8; arteriosclerosis *r* = 0.34, *p* = 0.001; IF/TA *r* = 0.08, *p* = 0.4).

**Figure 4:**
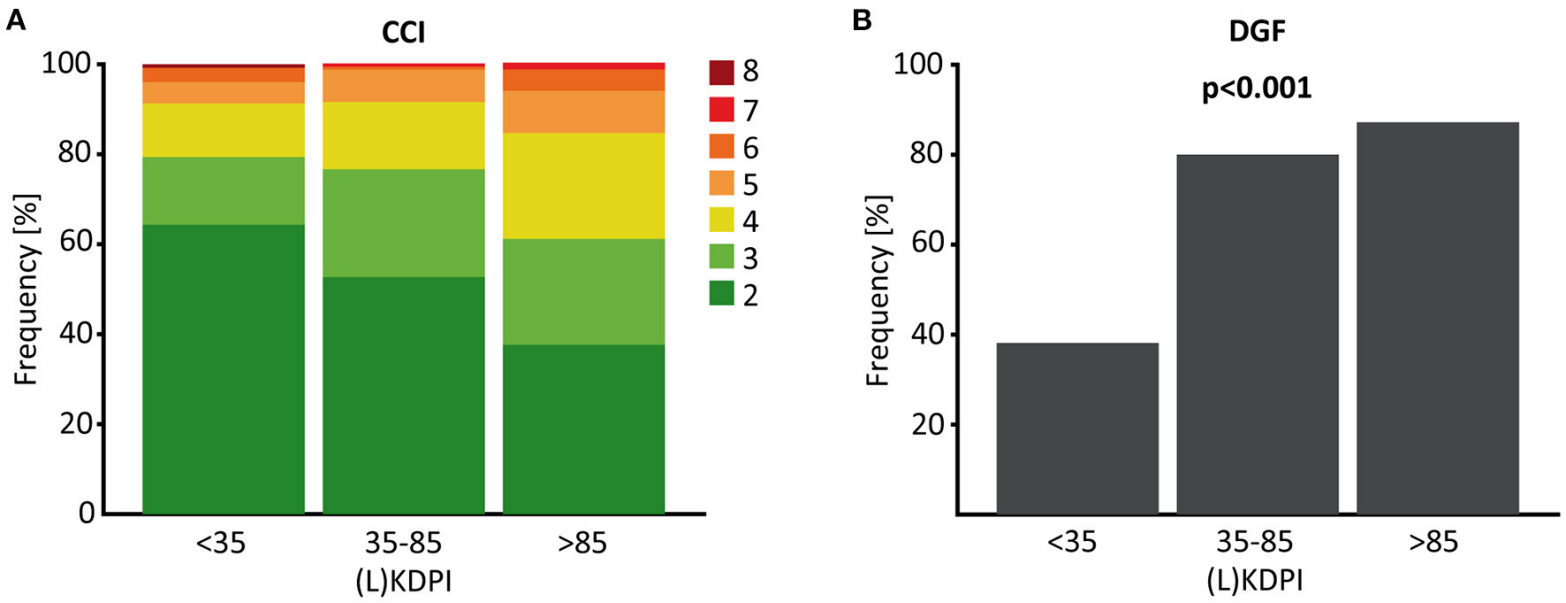
CCI and DGF depended on the (L)KDPI in living and deceased donation. **(A)** Percent stacked column chart of the Charlson Comorbidity Index (CCI) of living and deceased kidney transplantation with the (Living) Kidney Donor Profile Index [(L)KDPI] divided into <35, 35–85 and >85%. **(B)** Boxplot showing the percentage of delayed graft function (DGF) of kidney transplantations after living or deceased donation divided into (L)KDPI <35, 35–85, and >85%. Kruskal-Wallis test was used for calculation of *p*-value.

Furthermore, we found a moderate, but highly significant correlation between the (L)KDPI and the extent of ATI (*r* = 0.198, *p* < 0.001, Figure 3D). In line with this observation, higher rates of delayed graft function (DGF) could be revealed as clinical counterpart of severe ischemia-reperfusion injury (IRI) in transplants with a higher (L)KDPI (>35%) in contrast to a lower KDPI (<35%), as shown in Figure 4B.

Likewise, associations between the histopathological characteristics and the SCD/ECD-classification were apparent (Figures 3E–H). Glomerulosclerosis (η = 0.245, *p* < 0.001), arteriosclerosis (ϕ = 0.340, *p* < 0.001), and IF/TA (η = 0.161, *p* = 0.003) were significantly associated with the ECD status whereas ATI was not (ϕ = 0.104, *p* = 0.318). In living donations no statistically significant associations between chronic lesions except arteriosclerosis and no association between ATI and the ECD status existed (glomerulosclerosis η = 0.113, *p* = 0.277; arteriosclerosis ϕ = 0.395, *p* = 0.003; IF/TA η = 0.161, *p* = 0.119; ATI ϕ = 0.221, *p* = 0.205).

## Discussion

In this retrospective single center analysis, we evaluated the (L)KDPI against the background of the SCD/ECD-classification, which is more commonly used in Europe. We further compared this classification with baseline biopsies, which are routinely taken 10 min after the onset of reperfusion in our transplant center. The present study revealed the following major findings: *First*, the application of the KDPI and the LKDPI turned out to be a useful tool in this European single center analysis to assess the quality of donor kidneys all in line with earlier reports (12, 27). Furthermore, it was possible to demonstrate the comparability of living donation and DBD with KDPI and LKDPI. Most important, the (L)KDPI showed a distinct correlation with histopathological findings in baseline biopsies.

Interestingly, 48% of all ECD-kidneys in this study had a KDPI <85%. This underlines the usefulness of the 85% cutoff and together with the predictive value (C = 0.69) this suggests a better assessment of ECD kidneys by use of the (L)KDPI with regards to the further probable course after transplantation. Therefore, it can be assumed that the more complex and gradient (L)KDPI, which is based on a bigger range of information, reduces the risk to discard a valuable organ as compared to the dichotomous SCD/ECD-classification. On the other hand, the estimated overall graft survival of KDPI kidneys >85% of only about 60% after 5 years emphasizes the question if these organs should be used for transplantation. *Second*, this trial at hand proved that KDPI and LKDPI enable transplant physicians to compare graft quality between living and deceased donation in a non-US transplant cohort, the way the LKDPI classification was originally defined for (16). Hence, these data suggest an advantage of using the highly granular (L)KDPI to stratify the prognosis of donor kidneys origin as compared to the SCD/ECD-classification. However, several American and European validation studies on the discriminative ability of the KDPI it never exceeded a Harrell's C of 0.62–0.66 (10, 12, 13, 27, 28), which means that only 66% of the predictions hit the correct outcome. Accordingly, SCD-kidneys may be labeled by a KDPI >85% and be discarded. The rather high impact of donor age on transplant outcomes compared with the KDPI also underlines its additional predictive limits (29). Noteworthy, Bae et al. cautioned against an increasing mortality amongst patients remaining on dialysis and waiting for a kidney offer with a lower KDPI instead of transplantation of these kidneys (30). Fittingly, in our cohort, Assfalg et al. were able to demonstrate similar 5-year graft and patient survival after standard and rescue allocation (31). Interestingly, in the multivariate Cox-regression models of this analysis the KDPI and LKDPI turned out to have a predictive value whereas the ECD status, and cold ischemia time and recipient's age which are not included into the ECD-classification did not. On the other hand, a retrospective analysis of 5,667 patients older than 70 years showed a decreased relative risk of death of 0.75 in patients transplanted with ECD-kidneys as compared to patients remaining on the waiting list (32). ECD kidneys display a significant predictor of mortality in all age groups except for patients older than 70 years (33).

*Third*, the (L)KDPI in the investigated cohort correlated well with chronic lesions such as arteriosclerosis, glomerulosclerosis and IF/TA giving reason to expected lower graft quality of marginal donor kidneys, which was shown previously (34). This correlation was also observed in a study on pre-implantation biopsies (35). Kidney grafts can also be evaluated by pre-transplant donor biopsies, but due to a high heterogeneity in biopsy-technique, histological evaluation, and study design no valuable recommendation can be derived to include pre-transplant donor biopsies into daily routine (36). Nonetheless, Gandolfi et al. were able to show that pre-transplant donor biopsies allowed for save transplantation of high KDPI-kidneys provided that a specifically trained pathologist is available (37, 38). However, pre-transplant biopsies do not map renal ischemia reperfusion injury (IRI).

*Fourth*, a correlation between the (L)KDPI and the degree of ATI as histological hallmark of renal IRI is present in this cohort. Earlier studies demonstrated that marginal and especially ECD kidneys are significantly more vulnerable to cold ischemia time, which is part of the transplantation process after deceased donation and the subsequent tubular injury (39–41). Severe acute tubular injury becomes clinically apparent in delayed graft function (DGF) defined as dialysis in the first week after kidney transplantation (42). Gill et al. showed a decreased graft survival benefit of kidney transplantation with high KDPI grafts followed by DGF as compared to recipients of a higher quality graft followed by DGF but still better than in patients remaining on dialysis (43). DGF is a well-known independent risk factors of 1-year graft survival (44). Our data strongly underlines the approach that ATI could be a therapeutic target to improve graft quality of kidneys with high KDPI (45). The use of hypo- or normothermic machine perfusion may be an option here (46). Using *ex vivo* normothermic perfusion, Kabagambe et al. prompted increasing blood flow and urine output and histologically less ATI in 7 marginal kidneys with a mean KDPI of 79%, which were initially discarded for kidney transplantation based on a combination of clinical findings, suboptimal biopsies, long CIT, and/or poor hypothermic perfusion parameters (47).

Our study has several points for critical discussion. We investigated on a single center cohort with a moderate number of cases including kidney transplantation after deceased as well after living donation. Comparison of DBD to living donors bears a risk for bias due to big differences in organ quality (48). However, the LKDPI was developed to take these issues into account e.g., by negative values and was explicitly created for comparison of living to deceased donor grafts (16). Concerning DBD kidney grafts, outcomes might be biased by our selection policy accepting grafts from older donors with presumably lower quality for older recipients. Although the KDPI was predictive in a multivariate Cox-regression model including the recipients age, patients who received kidneys with a low KDPI were significantly younger than patients who received kidneys with a high KDPI (*p* < 0.001). Finally, the retrospective design of this study cannot reach the quality of a prospective observation study.

In conclusion, also in a European single center cohort the (L)KDPI for kidney transplants living donation and DBD is useful to assess organ quality more accurate than SCD/ECD-classification and to stratify their risk for later graft loss. Until this study there was no certainty, if the predictive value of the (L)KDPI translates into histopathological findings of baseline kidney biopsies. Additionally, the increase in Harrell's C after inclusion of IF/TA suggests an even better judgement of organ quality utilizing a biopsy. Thus, a prospective, multicenter study with a higher number of patients performing baseline biopsies is required to clarify, if the combination of the (L)KDPI and histopathological findings can improve the predicted outcome of kidney grafts. This might give clinicians the missing tool to better judge the value of grafts rendered as bad quality by current scores, since evidence proving the need to transplant these organs to improve patient survival accumulates (31, 49, 50). The overall small predictive value of the currently available tools illustrates the necessity for comprehensive international databases, further research on the predictive value of donor, graft, and transplant specific properties including more variables, and transplant physicians' courage to even accept marginal organs for distinct subgroups in times of growing organ shortage.

## Data Availability Statement

The raw data supporting the conclusions of this article are available from corresponding author upon reasonable request.

## Ethics Statement

The studies involving human participants were reviewed and approved by Ethics Committee of the Technical University of Munich, Germany. The patients/participants provided their written informed consent to participate in this study.

## Author Contributions

QB and SK wrote the manuscript. SK and CS designed the concept of the study and profile index of the study. QB, FH, CT, and SK collected clinical data. QB, FH, BH, and SK performed and reviewed statistical analysis. VA is responsible for the routinely performance of surgical biopsies used in this study. MB-H analyzed the biopsies. VA, KA, LR, and UH oversaw the study and critically discussed the manuscript. All authors approved the submitted version of the manuscript.

## Funding

This work was supported by the Klinikum Rechts der Isar of the Technical University of Munich.

## Conflict of Interest

The authors declare that the research was conducted in the absence of any commercial or financial relationships that could be construed as a potential conflict of interest.

## Publisher's Note

All claims expressed in this article are solely those of the authors and do not necessarily represent those of their affiliated organizations, or those of the publisher, the editors and the reviewers. Any product that may be evaluated in this article, or claim that may be made by its manufacturer, is not guaranteed or endorsed by the publisher.

## Abbreviations

ATI: acute tubular injury
CIT: cold ischemia time
DBD: donation after brainstem death
DGF: delayed graft function
ECD: expanded criteria donor
IF/TA: interstitial fibrosis and tubular atrophy
IRI: ischemia-reperfusion injury
KDPI: kidney donor profile index
KDRI: kidney donor risk index
LKDPI: living kidney donor profile index
(L)KDPI: (living)KDPI
OPTN: organ procurement and transplantation network
PRA: panel reactive antibodies
SCD: standard criteria donor.
